# Long-Term Survival Outcomes and Comparison of Different Treatment Modalities for Stage I-III Cervical Esophageal Carcinoma

**DOI:** 10.3389/fmed.2021.714619

**Published:** 2021-09-22

**Authors:** Yanwei Lu, Chenwang Xu, Haitao Wang, Tao Song, Shixiu Wu, Xiaodong Liang, Hong'en Xu

**Affiliations:** ^1^Department of Radiation Oncology, Oncology Center of Zhejiang Provincial People's Hospital, Affiliated People's Hospital, Hangzhou Medical College, Hangzhou, China; ^2^Department of Applied Physics, Hangzhou Medical College, Hangzhou, China; ^3^Department of Thoracic Surgery, Zhejiang Provincial People's Hospital, Affiliated People's Hospital, Hangzhou Medical College, Hangzhou, China; ^4^Department of Radiation Oncology, The Second Affiliated & Yuying Children's Hospital Wenzhou Medical University, Wenzhou, China

**Keywords:** cervical esophageal carcinoma, SEER, surgery, treatment modality, survival

## Abstract

**Purpose:** To investigate the survival outcomes, prognostic factors and treatment modalities of stage I-III cervical esophageal carcinoma (CEC) patients using data from the Surveillance, Epidemiology, and End Results (SEER) database from the period 2004–2016.

**Methods:** Patients with a histopathologic diagnosis of CEC were included. The primary endpoint was overall survival (OS). Univariate and multivariate analyses of OS were performed using Cox proportional hazards models, and OS was compared using the Kaplan-Meier method and log-rank test.

**Results:** A total of 347 patients in the SEER database were enrolled. The median OS was 14.0 months, with a 5-year OS rate of 20.9%. The parameters that were found to significantly correlate with OS in the multivariate analysis were age at diagnosis [*P* < 0.001, hazard ratio (HR) = 1.832], sex [*P* < 0.001, HR= 1.867], histology [*P* = 0.001, HR = 0.366], surgery at the primary site [*P* = 0.021, HR = 0.553], radiotherapy (RT, *P* = 0.017, HR = 0.637) and chemotherapy (CT, *P* < 0.001, HR = 0.444). Comparison among the three treatment modalities demonstrated that a triple therapy regimen consisting of surgery, RT and CT was associated with a longer survival time than the other two treatment modalities before and after propensity score matching (PSM). However, triple therapy showed no significant survival benefit over double therapy (*P* = 0.496 before PSM and *P* = 0.184 after PSM).

**Conclusions:** The survival of patients with CEC remains poor. Surgery, RT and CT were all strongly correlated with OS. We recommend a triple therapy regimen for select CEC patients based on the findings of the current study, although this recommendation should be further confirmed by prospective studies with large sample sizes.

## Introduction

Cervical esophageal cancer (CEC), which develops between the lower border of the cricoid cartilage and the thoracic inlet, is an uncommon but deadly type of esophageal carcinoma, accounting for more than 5% of all cases, and the major histotype is squamous cell carcinoma (SCC) (1, 2). Due to its malignant biological behavior and failure to produce early symptoms, CEC often presents as local-regional disease or locally advanced disease at diagnosis (3).

Historically, surgical resection has played a pivotal role in the treatment of esophageal cancer, especially for middle- or lower-third tumors (4–6). For CEC, earlier series of retrospective studies also provided evidence to support radical resection, including pharyngo-laryngo-esophagectomy (PLE), in this challenging field (7–9). However, the intimate relationship between the cervical esophagus and vital anatomical local structures such as the hypopharynx, trachea, larynx, thyroid, and recurrent laryngeal nerves precludes the widespread application of a surgical approach (10, 11). Thus, non-surgical treatment, including definitive concurrent chemoradiotherapy (CTRT), has been recommended as an alternative treatment strategy for CEC. Unfortunately, due to its rarity, high-level evidence from randomized controlled trials on the management of CEC is still lacking. The landmark Radiation Therapy Oncology Group (RTOG) 8,501 trial compared the efficacy of CTRT with radiotherapy (RT) alone (single therapy) for non-metastatic, thoracic esophageal SCC, which resulted in a 5-year overall survival (OS) rate of 14–26% in the CTRT group vs. 0% in the single therapy group (12, 13). Given these findings and the results of a series of studies reporting a comparable OS rate for CEC patients treated with CTRT (14–17), both the National Comprehensive Cancer Network and European Society for Medical Oncology guidelines currently recommend CTRT for the management of CEC.

As surgical techniques have evolved, surgeons have attempted to apply minimally invasive strategies instead of mutilating resections to improve the dismal survival outcome of CEC (18–20). Additionally, understanding how different prognostic factors influence survival will lead to better management of CEC. Thus, this population-based study aimed to explore the long-term survival outcomes and potential prognostic factors of OS in CEC patients, to evaluate the efficacy of triple therapy consisting of surgical resection and CTRT, and to compare this efficacy with that of single (RT alone) and double therapy (CTRT) using data from the National Cancer Institute's Surveillance, Epidemiology, and End Results (SEER) database.

## Materials and Methods

### Data Sources and Inclusion Criteria

We obtained permission to access the data registered in the database for the purpose of research only (Reference number: 10579-Nov2019) by using SEER^*^Stat version 8.3.6. The Institutional Review Board (IRB) of Zhejiang Provincial People's Hospital determined that the data in this dataset contained no personal identifiers and were publicly available after permission. Therefore, formal IRB review was waived. We affirm that the methods in this study were performed in accordance with the Declaration of Helsinki.

In this study, the International Statistical Classification of Diseases for Oncology, 3rd Edition (ICD-O-3), site code C15.0 (cervical esophagus) was applied to extract data in the SEER program. The time span was from 2004 to 2016. The year 2004 was selected as the first year of study given that the 6th edition of clinical/TNM staging has been uniformly used in SEER since 2004 and because studies have demonstrated the superior prognostic impact of the 6th edition over the later version of TNM staging for esophageal cancer patients who received CTRT (21, 22). For patients diagnosed in 2016, the tumors were also restaged based on the definition of the 6th edition of TNM staging.

**Figure 1:**
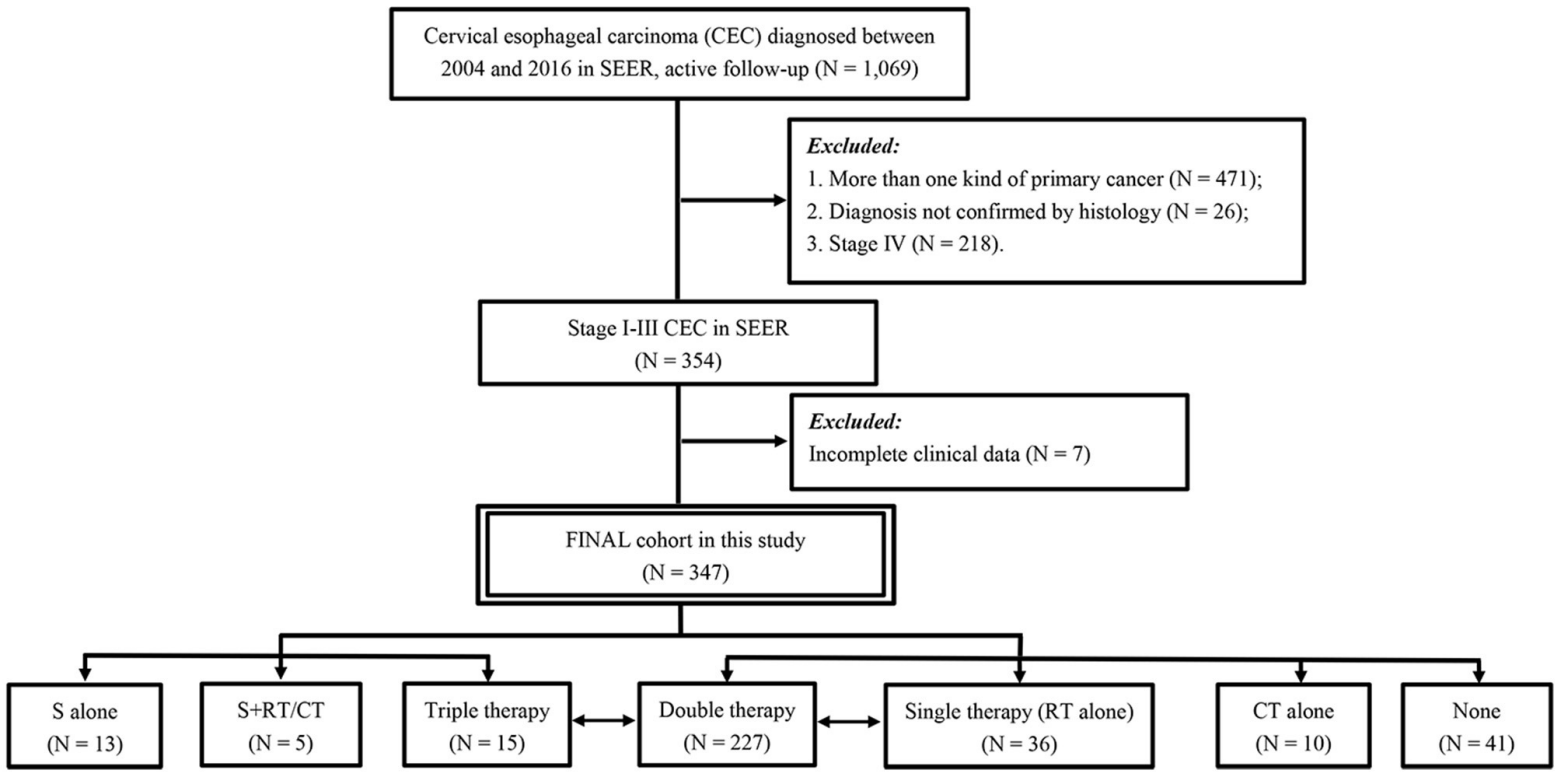
Patient selection flowchart.

Patients were chosen according to the following criteria: (1) histopathological diagnosis of CEC between 2004 and 2016 and (2) primary diagnosis of CEC. The main exclusion criteria were as follows: (1) more than one primary cancer, with CEC not being the first diagnosed cancer; (2) stage IV disease; and (3) incomplete clinical data in the database (Figure 1).

### Statistical Analysis

The following variables were collected from the SEER database: marital status, age at diagnosis, race, sex, tumor grade, tumor histology, TNM stage, surgery at the primary site, surgery at regional lymph nodes, RT, RT sequence with surgery, chemotherapy (CT), tumor size, SEER cause-specific death classification, survival time in months and survival status (alive or dead). The SEER items for cause-specific death classification indicate whether the person died of the cancer or of causes other than CEC.

Binary logistic regression was performed to identify the potential factors leading to surgical treatment based on the design of the current study, with calculation of the odds ratio (OR) and 95% confidence intervals (CI). The chi-square test or Fisher's exact test was employed to analyze the differences in baseline characteristics among the three different treatment modalities as appropriate. OS was defined as the interval from the diagnosis of CEC to death from any cause or the last follow-up registered in the database. Disease-specific survival (DSS) was defined as the interval from the diagnosis of cancer to death caused by CEC or the last follow-up. Survival associated with different parameters was estimated using the Kaplan-Meier method, and survival times were compared using the log-rank test. A univariate analysis was performed to identify the predictive factors for OS. Variables identified with a *P*-value ≤ 0.05 in the univariate analysis were incorporated into the multivariate analysis. Multivariate analysis of the predictive factors for OS was estimated using a Cox regression model, with hazard ratios (HRs) and 95% CIs. In addition, to reduce the imbalance of potential confounders among the three different treatment modalities, propensity score matching (PSM) was performed at a 1:1:1 ratio. The matched covariates included age at diagnosis, marital status, race, sex, histology, differentiation, tumor size, clinical stage, T stage and N stage. The method for PSM has been described previously (23). All statistical analyses were conducted using R software version 3.6.2 (https://www.r-project.org) and IBM SPSS statistical software package version 25.0 (SPSS, Armonk, New York, USA), and the survival curves were drawn with GraphPad Prism 8.0 (GraphPad Software, San Diego, CA, USA). Differences were considered significant if the two-sided *P*-values were < 0.05.

## Results

### Patient Characteristics

**Figure 2:**
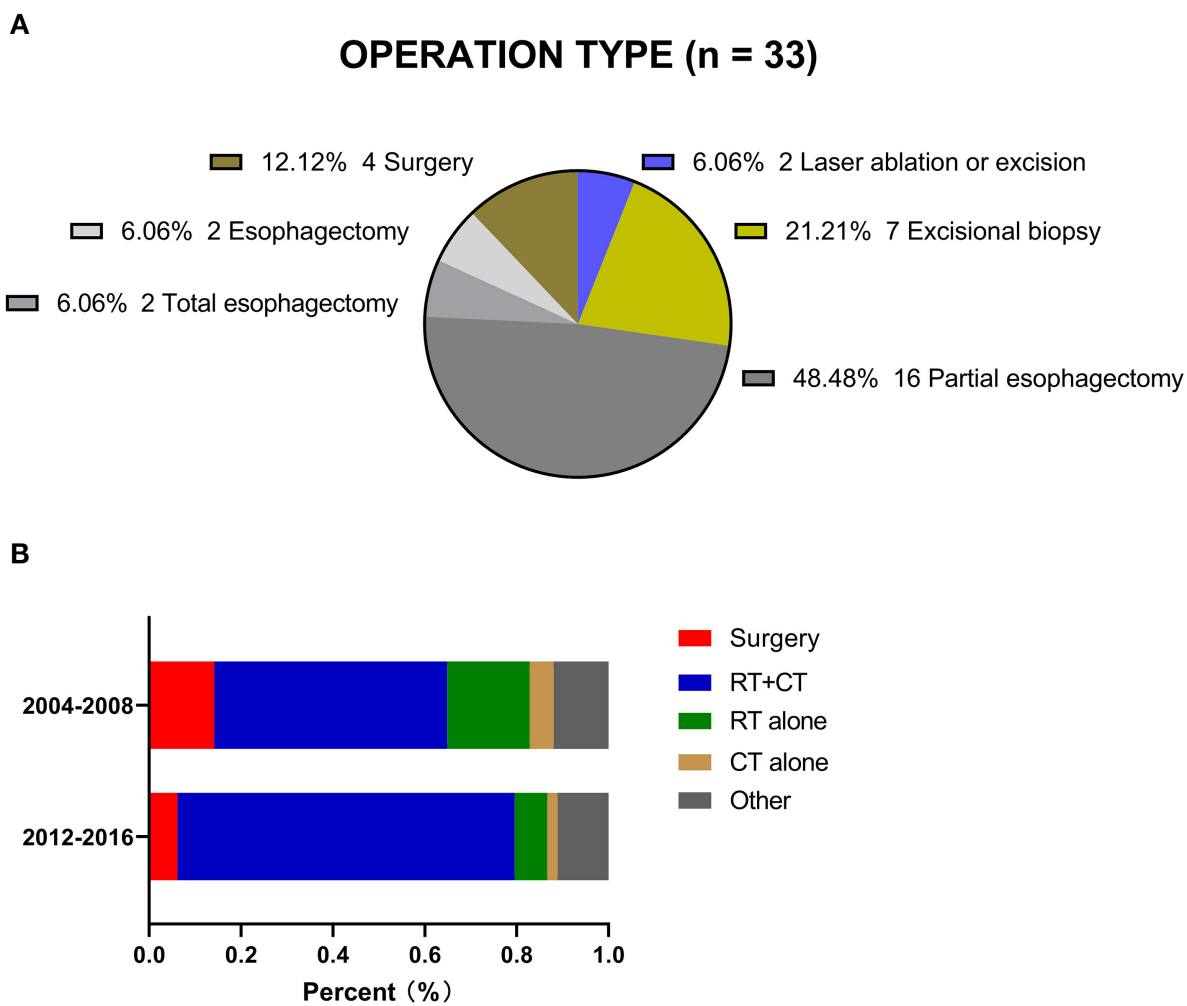
**(A)** Operation type registered in the SEER. **(B)** Two treatment periods for CEC patients in the SEER.

The study sample consisted of 347 stage I-III CEC patients diagnosed from 2004 to 2016 (Table 1). The median age at diagnosis was 65 years (IQR, 57–74 years). A total of 265 patients were white (76.4%), and more than half of the included patients were male (63.1%). The major histology type was SCC (92.2%), and the median tumor size was 41 millimeters. The proportions of patients with stage I, II and III CEC were 18.7, 28.8, and 52.5%, respectively. Thirty-three (9.5%) patients in this cohort underwent cancer-directed surgery at the primary site (Figure 2A). Among all these patients, 20 (60.6%) underwent at least partial esophagectomy, nine (27.3%) patients received postoperative RT, and seven (21.2%) underwent neoadjuvant RT followed by local surgery. A total of 254 (73.2%) patients received chemotherapy (CT) in this cohort.

**Table 1:** Baseline characteristics of the CEC patients.

**Characteristic**	**Frequency, (%)**
**Age (years)**	
Median (IQR)	65 (57-74)
**Marital status**	
Married	158 (45.5)
Unmarried and others	189 (54.5)
**Race**	
White	265 (76.4)
Non-white	82 (23.6)
**Sex**	
Female	128 (36.9)
Male	219 (63.1)
**Histology**	
SCC	320 (92.2)
Non-SCC	27 (7.8)
**Differentiation**	
Well or fairly differentiated	181 (52.1)
Poorly or undifferentiated	88 (25.4)
Unknown	78 (22.5)
**Tumor size (mm)**	
<41	116 (33.4)
≥41	117 (33.7)
Unknown	114 (32.9)
**Clinical stage (AJCC 2002)**	
Stage I	65 (18.7)
Stage II	100 (28.8)
Stage III	182 (52.5)
**T stage**	
T_1−2_	116 (33.4)
T_3−4_	231 (66.6)
**N stage**	
Negative	177 (51.0)
Positive	170 (49.0)
**Surgery at the primary site**	
No	314 (90.5)
Surgery	33 (9.5)
**Surgery at regional lymph nodes**	
No/unknown	315 (90.8)
Yes	32 (9.2)
**Radiotherapy (RT)**	
No/unknown	66 (19.0)
Yes	281 (81.0)
Delivery of radiotherapy^*^	
EBRT	273 (97.2)
Not specified	8 (2.8)
**Chemotherapy (CT)**	
No/unknown	93 (26.8)
Yes	254 (73.2)

*IQR, interquartile range; SCC, squamous cell carcinoma; AJCC, American Joint Committee on Cancer;*

* *analyzed within patients who received RT; EBRT, external beam RT*.

The proportion of patients who underwent surgery decreased from 14.2% during 2004–2008 to 6.2% during 2012–2016. The proportion of patients treated with RT and CT increased from 50.7% to 73.4% over the same time frame. The proportion of patients who received other forms of treatment remained relatively constant, at 11.1–11.9%, although there was no detailed information in the database (Figure 2B).

Multivariate analysis demonstrated that age at diagnosis (<65 vs. ≥ 65, *P* = 0.037, OR = 0.405), marital status (married vs. unmarried and others, *P* = 0.006, OR = 0.296), tumor histology (SCC vs. non-SCC, *P* = 0.001, OR = 6.004) and tumor size (<41 vs. ≥ 41, *P* = 0.040, OR = 0.358; <41 vs. unknown, *P* = 0.016, OR = 0.271) were significantly associated with surgical treatment (Supplementary Table 1).

### Prognostic Factors for OS

The median OS of the overall population was 14.0 months (95% CI: 11.971–16.029). The 3- and 5-year OS rates were 29.1 and 20.9%, respectively. A total of 254 (73.2%) CEC patients died during the follow-up period. Among them, 220 (86.6%) patients died due to CEC directly. The median DSS was 16.0 months (95% CI: 12.542–19.458), with 3- and 5-year DSS rates of 33.7 and 27.4%, respectively. The survival curve indicated a non-significant relationship between OS and DSS (*P* = 0.112, Supplementary Figure 1). Based on this result, further univariate and multivariate analyses were performed to determine the factors correlated with OS.

**Figure 3:**
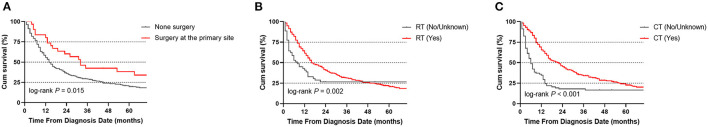
Overall survival (OS) of CEC patients according to whether they underwent surgery **(A)**, RT **(B)**, or CT **(C)**.

Several covariates were significantly associated with OS (Table 2) according to the univariate log-rank test results, including age at diagnosis (*P* < 0.001), marital status (*P* = 0.005), sex (*P* = 0.005), tumor histology (*P* = 0.005), surgery at the primary site (*P* = 0.018), use of RT (*P* = 0.002) and use of CT (*P* < 0.001). In the multivariate analysis, the factors significantly associated with OS were age at diagnosis (<65 vs. ≥ 65 years, *P* < 0.001, HR = 1.832), sex (*P* < 0.001, HR = 1.867), tumor histology (*P* = 0.001, HR = 0.366), surgery at the primary site (*P* = 0.021, HR = 0.553), use of RT (*P* = 0.017, HR = 0.637) and use of CT (*P* < 0.001, HR = 0.444; Figure 3).

**Table 2:** Univariate and multivariate analyses of overall survival (OS).

	**overall survival (OS)**							
	**Univariate**				**Multivariate**			
	***P*** **-value**	**HR**	**95% CI**	**95% CI**	***P*** **-value**	**HR**	**95% CI**	**95% CI**
**Factor**			**Lower**	**Upper**			**Lower**	**Upper**
**Age at diagnosis (years)**								
<65	Reference				Reference			
≥65	<0.001	2.051	1.593	2.642	<0.001	1.832	1.417	2.370
**Marital status**								
Married	Reference				Reference			
Unmarried and others	0.050	1.283	1.000	1.647	0.068	1.275	0.983	1.655
**Race**								
White	Reference				–			
Non-white	0.051	1.320	0.999	1.743				
**Sex**								
Female	Reference				Reference			
Male	0.005	1.466	1.125	1.911	<0.001	1.867	1.417	2.459
**Histology**								
SCC	Reference				Reference			
Non-SCC	0.005	0.465	0.271	0.798	0.001	0.366	0.199	0.672
**Differentiation**								
Well or fairly differentiated	Reference				–			
Poorly or undifferentiated	0.819	0.966	0.717	1.301				
Unknown	0.159	0.795	0.578	1.094				
**Tumor size (mm)**								
<41	Reference				–			
≥41	0.135	1.260	0.931	1.707				
Unknown	0.265	1.189	0.877	1.611				
**Clinical stage (AJCC 2002)**								
Stage I-II	Reference				–			
Stage III	0.168	1.190	0.929	1.523				
**T stage**								
T_1−2_	Reference				–			
T_3−4_	0.872	1.021	0.788	1.323				
**N stage**								
Negative	Reference				–			
Positive	0.067	1.261	0.984	1.616				
**Surgery at the primary site**								
None	Reference				Reference			
Yes	0.018	0.583	0.372	0.913	0.021	0.553	0.334	0.916
**Regional lymphadenectomy**								
None/unknown	Reference				–			
Yes	0.184	0.748	0.487	1.148				
**Radiotherapy**								
None/unknown	Reference				Reference			
Yes	0.002	0.620	0.455	0.845	0.017	0.637	0.439	0.924
**Chemotherapy**								
None/unknown	Reference				Reference			
Yes	<0.001	0.473	0.362	0.618	<0.001	0.444	0.325	0.607

*HR, hazard ratio; CI, confidence interval*.

### Comparison of the Three Different Treatment Modalities Before and After PSM

As local therapy has remained the mainstay of treatment for the management of stage I-III CEC patients, we further divided this cohort of patients into three subgroups: (1) single therapy group: patients who received RT alone (*N* = 36); (2) double therapy group: patients who received RT combined with CT (non-surgical group) (*N* = 227); and (3) triple therapy group: patients who received surgery combined with RT and CT (*N* = 15). The baseline characteristics of the three groups before and after PSM are shown in Supplementary Table 2. Among the 15 patients who received trimodal therapy, 12 (80.0%) patients underwent at least partial esophagectomy, and one patient underwent excisional biopsy, but the surgical treatment of the other two patients was not detailed in the records. In addition, 6 patients received postoperative radiotherapy, and 7 patients received preoperative radiotherapy, but the treatment sequence for the other 2 patients was unknown.

**Figure 4:**
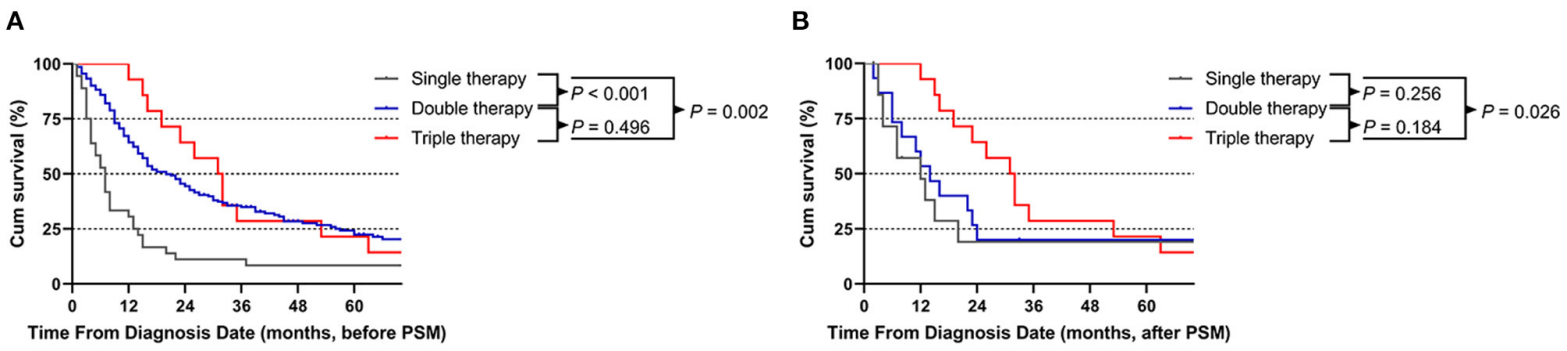
Comparison of patients who underwent the three different treatment modalities before **(A)** and after **(B)** propensity score matching (PSM) analysis.

The median OS times of single therapy, double therapy and triple therapy before PSM were 7, 20, and 31 months, respectively. Patients receiving triple therapy had the longest survival time (double therapy vs. single therapy, *P* < 0.001; triple therapy vs. single therapy; *P* = 0.002). However, triple therapy showed a non-significant survival benefit over double therapy (*P* = 0.496; Figure 4A). After PSM, the median OS times for patients receiving single therapy, double therapy and triple therapy were 7, 14, and 31 months, respectively. Similarly, the patients receiving triple therapy had the longest survival time, with a significant difference between those receiving single therapy and those receiving triple therapy (*P* = 0.026; Figure 4B).

Multivariate analysis of the different treatment modalities before PSM revealed that age at diagnosis (*P* < 0.001, HR = 1.727), sex (*P* < 0.001, HR = 1.776) and treatment modality (single therapy vs. double therapy, *P* < 0.001, HR = 0.471; single therapy vs. triple therapy, *P* = 0.028, HR = 0.399) were significant prognostic factors for OS, but this difference was observed only between single therapy and triple therapy (*P* = 0.038, HR = 0.410) after PSM (Supplementary Table 3).

## Discussion

The present analysis represents one of the largest studies to date exploring the treatment modalities, survival outcomes and prognostic factors of stage I-III CEC patients. When we compared our study results with those from a recently published retrospective study evaluating 500 Chinese CEC patients, we found a similar distribution of baseline characteristics between Western and Eastern countries (24). The proportion of patients receiving surgical resection in this unique setting was in accordance with a report from the Netherlands (25) that included 2,783 patients diagnosed with proximal esophageal cancers. The data revealed that surgical resection was performed in 17% of patients during 1989–1994 and in only 2% during 2010–2014. In contrast, the proportion of patients treated with CTRT increased from 1% during 1989–1994 to 49% during 2010–2014. Unclear survival benefits and impaired quality of life (QoL) brought about by mutilating resections were considered the main factors explaining this tendency, which raise the following question: Does surgery have a role in the management of CEC in the contemporary era?

To illustrate this controversial situation, we first explored the value of double therapy, especially CTRT, for the management of CEC in the literature. A 2007 retrospective analysis from Canada reviewed 71 CEC patients who were treated with CTRT between 1997 and 2005. The survival analysis showed that the median OS time was 15 months with a 5-year OS rate of 21% for the whole group (26). Ten years later, the authors further updated their single-institution experience of CTRT for the management of CECs. Although the concurrent regimens/doses of CT, the techniques for the delivery of RT, and the dose of RT (such as three-dimensional conformal RT to intensity-modulated RT) changed over time, the survival result still demonstrated a similar 5-year OS rate of 28% among the 81 CEC patients (2). Another large-sample, retrospective study from the National Cancer Data Base (NCDB) reported similar results. In that study, 707 stage I-III CEC patients who received CTRT and had available survival results were enrolled. Despite different radiation doses delivered to the CEC, the 5-year OS rate was ~25% (27). Combined with the data from the aforementioned retrospective studies (14, 15, 17) and the subgroup analysis in the current report, it seems that double therapy has reached its plateau. Additionally, a full/higher dose CTRT has been associated with notable complications such as myelosuppression, severe mucositis, pharyngitis, dermatitis, hypothyroidism/hypoparathyroidism, esophageal stricture, and carotid blowout (18). Thus, new therapeutic combinations for the better management of CEC are desperately needed.

Instead of large-area mutilating resections, minimally invasive surgery techniques such as PLE and larynx-preserving surgery have revolutionized surgical treatment options for CEC over the last two decades (8, 11, 28). In 2016, Saeki et al. reviewed their single-center experience describing the role of surgery in the treatment of CEC (29). A total of 63 consecutive CEC patients who received CTRT followed by resection were analyzed, and the clinical results were compared with those of 977 non-CEC patients from 1980 to 2013. With the advancement of surgical techniques, minimally invasive approaches such as thoracoscopic surgery and microscopic venous anastomosis are currently the main procedures used at their institution (30). Short-term results showed that there were no differences in the frequency of all postoperative complications according to the tumor location. Long-term survival analysis further indicated that compared with the OS results obtained with data from 1980 to 1999, a significant improvement was observed in CEC patients who underwent minimally invasive surgery from 2000 to 2013 (71.5 vs. 40.7%, *P* = 0.003). Other minimally invasive approaches, including total PLE (31), larynx-preserving hybrid surgery with endoscopic laryngopharyngeal surgery and open surgery (32) and larynx-preserving surgery (33), were also attempted, and satisfactory 2-year OS rates of 64.3, 71.5, and 50.6%, respectively, were achieved. In addition, primary RT and surgery have been directly compared in a large retrospective study (34). In that study, 63 patients were enrolled in the surgery group, and 161 patients were enrolled in the RT with or without CT group. Survival analysis demonstrated that the 2-year OS rates were 49.3 and 50.7% for patients undergoing primary RT and surgery (*P* = 0.31), respectively. Even when comparing matched patients between the two groups, the survival difference was non-significant. Considering the almost equal distribution of patients receiving preoperative RT or postoperative RT in the triple therapy group registered in SEER, the optimal sequence of primary RT/CTRT or surgery warrants further investigation.

Another important concern regarding the use of surgery or CTRT is QoL. Previously, a retrospective study with a small sample size indicated that both treatment modalities could effectively improve QoL compared with baseline. For patients undergoing surgery, the preoperative and postoperative QoL scores were 72.73 ± 1.22 and 74.27 ± 1.83, respectively (*P* = 0.003), and the changes in QoL scores in the surgery group seemed to be better than those in the CTRT group, although the difference was not significant (35). Meanwhile, Makino et al. evaluated the efficiency of larynx-preserving (LP) esophagectomy vs. non-preservation (NP) procedures to preserve organ function and improve QoL in 100 CEC patients (36). Compared with the NP group, the LP group showed significant improvement in the 5-year OS and DSS rates (OS: 51.2 vs. 36.3%, *P* = 0.0006; DSS: 44.1 vs. 28.1%, *P* = 0.0092, respectively), but no significant difference in overall postoperative complications (*P* = 0.265) or hospital stays (*P* = 0.372) was observed. Furthermore, regarding salvage surgical treatment after CTRT, a retrospective analysis compared the safety and efficiency of salvage total pharyngolaryngectomy and cervical esophagectomy (TPLCE) between 11 CEC patients and 26 hypopharyngeal cancer patients (37). Significantly more CEC patients had trachea-related complications after salvage TPLCE than hypopharyngeal cancer patients. Although salvage TPLCE offers a chance of prolonged survival, the balance between curability and safety, especially QoL, must be maintained.

It is essential to acknowledge that some questions remain unanswered. First, this was a retrospective analysis of data registered in the SEER database. After patient selection, only 15 CEC patients in the triple therapy group and 36 patients in the single therapy group were included for comparison with the double therapy group, which consisted of 227 patients. Although PSM was employed to limit confounding factors among different treatment modalities, the small sample size of patients, especially in the triple therapy group, significantly limits the statistical power of this comparison. Other drawbacks include unavailability of data for some variables, such as treatment toxicities, surgical complications, the sequence of trimodal therapy, specific CT regimens, treatment responses and QoL. Therefore, there is potential bias, which might influence the final conclusions; these findings should be externally verified in future large-sample studies.

## Conclusions

Based on the findings of the current study, compared with single RT therapy, both double therapy and triple therapy yielded favorable survival outcomes for stage I-III CEC patients. Furthermore, although the difference between CTRT and the triple therapy regimen including surgical resection was not significant, given the advancements in surgical procedures, we recommend surgical resection after careful evaluation in combination with RT and CT for select CEC patients in future oncological practice.

## Data Availability Statement

The original contributions presented in the study are included in the article/Supplementary Material, further inquiries can be directed to the corresponding author/s.

## Ethics Statement

Ethical review and approval was not required for the study on human participants in accordance with the local legislation and institutional requirements. Written informed consent for participation was not required for this study in accordance with the national legislation and the institutional requirements.

## Author Contributions

YL: conception and design, drafting, final approval, and accountable for relevant aspects. CX and HW: provision of study materials, collection and assembly of data, drafting of the manuscript, final approval, and accountable for relevant aspects. TS: conception and design, provision of study materials, collection and assembly of data, drafting of the manuscript, final approval, and accountable for relevant aspects. SW: conception and design, revision, final and approval, and accountable for relevant aspects. XL and HX: data analysis and interpretation, drafting of the manuscript, final approval, and accountable for relevant aspects. All authors contributed to the article and approved the submitted version.

## Funding

This work was funded by a Grant from the Medical Science and Technology Project of Zhejiang Province, China (No. 2021KY510) and a Grant from the CSCO-SciClone Pharmaceuticals, China (Y-2020Sciclone/qn-0075). The funders had no role in the study design, analysis, writing or submission.

## Conflict of Interest

The authors declare that the research was conducted in the absence of any commercial or financial relationships that could be construed as a potential conflict of interest.

## Publisher's Note

All claims expressed in this article are solely those of the authors and do not necessarily represent those of their affiliated organizations, or those of the publisher, the editors and the reviewers. Any product that may be evaluated in this article, or claim that may be made by its manufacturer, is not guaranteed or endorsed by the publisher.
