# Supplementary material for: Long-Term Survival Outcomes and Comparison of Different Treatment Modalities for Stage I-III Cervical Esophageal Carcinoma
Source: Front Med (Lausanne). 2021 Sep 22;8:714619. doi: 10.3389/fmed.2021.714619 (PMC8492900; doi:10.3389/fmed.2021.714619)
Supplement: Supplementary file 1 [file Table_1.docx]

Table S1. Univariate and multivariate analyses of surgical treatment.

|  | Surgical treatment | | | | | | | | |
| --- | --- | --- | --- | --- | --- | --- | --- | --- | --- |
|  | Univariate | | | |  | Multivariate | | | |
| *Factor* | ***P*** *value* | OR | *95% CI* | *95% CI* |  | ***P*** *value* | OR | *95% CI* | *95% CI* |
|  |  |  | *Lower* | *Upper* |  |  |  | *Lower* | *Upper* |
| *Age at diagnosis (years)* |  |  |  |  |  |  |  |  |  |
| < 65 | Reference |  |  |  |  | Reference |  |  |  |
| ≥ 65 | 0.013 | 0.373 | 0.172 | 0.810 |  | 0.037 | 0.405 | 0.173 | 0.945 |
| *Marital status* |  |  |  |  |  |  |  |  |  |
| Married | Reference |  |  |  |  | Reference |  |  |  |
| Unmarried and others | 0.002 | 0.279 | 0.126 | 0.620 |  | 0.006 | 0.296 | 0.125 | 0.699 |
| *Race* |  |  |  |  |  |  |  |  |  |
| White | Reference |  |  |  |  | - |  |  |  |
| Nonwhite | 0.441 | 0.696 | 0.277 | 1.749 |  |  |  |  |  |
| *Sex* |  |  |  |  |  |  |  |  |  |
| Female | Reference |  |  |  |  | - |  |  |  |
| Male | 0.119 | 1.993 | 0.845 | 4.424 |  |  |  |  |  |
| *Histology* |  |  |  |  |  |  |  |  |  |
| SCC | Reference |  |  |  |  | Reference |  |  |  |
| Non-SCC | < 0.001 | 7.596 | 3.123 | 18.476 |  | 0.001 | 6.004 | 2.163 | 16.665 |
| *Differentiation* |  |  |  |  |  |  |  |  |  |
| Well or fairly differentiated | Reference |  |  |  |  | - |  |  |  |
| Poorly or undifferentiated | 0.509 | 0.737 | 0.298 | 1.825 |  |  |  |  |  |
| Unknown | 0.709 | 0.841 | 0.338 | 2.089 |  |  |  |  |  |
| *Tumor size (mm)* |  |  |  |  |  |  |  |  |  |
| < 41 | Reference |  |  |  |  | Reference |  |  |  |
| ≥ 41 | 0.040 | 0.400 | 0.166 | 0.960 |  | 0.040 | 0.358 | 0.134 | 0.954 |
| Unknown | 0.027 | 0.356 | 0.143 | 0.889 |  | 0.016 | 0.271 | 0.094 | 0.784 |
| *Clinical stage (AJCC 2002)* |  |  |  |  |  |  |  |  |  |
| Stage I-II | Reference |  |  |  |  | Reference |  |  |  |
| Stage III | 0.024 | 0.418 | 0.196 | 0.891 |  | 0.593 | 1.451 | 0.371 | 5.680 |
| *T stage* |  |  |  |  |  |  |  |  |  |
| T_1-2_ | Reference |  |  |  |  | Reference |  |  |  |
| T_3-4_ | 0.023 | 0.433 | 0.210 | 0.893 |  | 0.194 | 0.448 | 0.134 | 1.504 |
| *N stage* |  |  |  |  |  |  |  |  |  |
| Negative | Reference |  |  |  |  | Reference |  |  |  |
| Positive | 0.011 | 0.356 | 0.161 | 0.791 |  | 0.027 | 0.311 | 0.110 | 0.877 |

OR, odds ratio; CI, confidence interval.
