# Supplementary material for: Long-Term Survival Outcomes and Comparison of Different Treatment Modalities for Stage I-III Cervical Esophageal Carcinoma
Source: Front Med (Lausanne). 2021 Sep 22;8:714619. doi: 10.3389/fmed.2021.714619 (PMC8492900; doi:10.3389/fmed.2021.714619)
Supplement: Supplementary file 2 [file Table_2.docx]

Table S2. Baseline characteristics before and after propensity score matching (PSM) analysis among the patients who underwent the three different treatment modalities.

|  | Before PSM | | | | | | | | |  | | After PSM | | | | | | | | | |  |
| --- | --- | --- | --- | --- | --- | --- | --- | --- | --- | --- | --- | --- | --- | --- | --- | --- | --- | --- | --- | --- | --- | --- |
| Characteristic | N |  | Triple therapy^‡^ (%) |  | Double therapy (%) |  | Single therapy (%) |  | *P* value | |  | | N |  | Triple therapy (%) |  | Double therapy (%) |  | Single therapy (%) |  | P value | |
| *Age (years)* |  |  |  |  |  |  |  |  | 0.004 | |  | |  |  |  |  |  |  |  |  | 0.779* | |
| age < 65 | 142 |  | 11 (73.3) |  | 121 (53.3) |  | 10 (27.8) |  |  | |  | | 31 |  | 11 (73.3) |  | 11 (73.3) |  | 9 (60.0) |  |  | |
| age ≥ 65 | 136 |  | 4 (26.7) |  | 106 (46.7) |  | 26 (72.2) |  |  | |  | | 14 |  | 4 (26.7) |  | 4 (26.7) |  | 6 (40.0) |  |  | |
| *Marital status* |  |  |  |  |  |  |  |  | 0.057 | |  | |  |  |  |  |  |  |  |  | 0.063 | |
| Married | 121 |  | 11 (73.3) |  | 95 (41.9) |  | 15 (41.7) |  |  | |  | | 22 |  | 11 (73.3) |  | 5 (33.3) |  | 6 (40.0) |  |  | |
| Unmarried and others | 157 |  | 4 (26.7) |  | 132 (58.1) |  | 21 (58.3) |  |  | |  | | 23 |  | 4 (26.7) |  | 10 (66.7) |  | 9 (60.0) |  |  | |
| *Race* |  |  |  |  |  |  |  |  | 0.433* | |  | |  |  |  |  |  |  |  |  | 0.894* | |
| White | 212 |  | 13 (86.7) |  | 174 (76.7) |  | 25 (69.4) |  |  | |  | | 36 |  | 13 (86.7) |  | 11 (73.3) |  | 12 (80.0) |  |  | |
| Nonwhite | 66 |  | 2 (13.3) |  | 53 (23.3) |  | 11 (30.6) |  |  | |  | | 9 |  | 2 (13.3) |  | 4 (26.7) |  | 3 (20.0) |  |  | |
| *Sex* |  |  |  |  |  |  |  |  | 0.572 | |  | |  |  |  |  |  |  |  |  | 0.894* | |
| Female | 99 |  | 4 (26.7) |  | 84 (37.0) |  | 11 (30.6) |  |  | |  | | 9 |  | 4 (26.7) |  | 2 (13.3) |  | 3 (20.0) |  |  | |
| Male | 179 |  | 11 (73.3) |  | 143 (63.0) |  | 25 (69.4) |  |  | |  | | 36 |  | 11 (73.3) |  | 13 (86.7) |  | 12 (80.0) |  |  | |
| *Histology* |  |  |  |  |  |  |  |  | 0.072* | |  | |  |  |  |  |  |  |  |  | 0.594* | |
| SCC | 262 |  | 12 (80.0) |  | 215 (94.7) |  | 35 (97.2) |  |  | |  | | 40 |  | 12 (80.0) |  | 14 (93.3) |  | 14 (93.3) |  |  | |
| Non-SCC | 16 |  | 3 (20.0) |  | 12 (5.3) |  | 1 (2.8) |  |  | |  | | 5 |  | 3 (20.0) |  | 1 (6.7) |  | 1 (6.7) |  |  | |
| *Differentiation* |  |  |  |  |  |  |  |  | 0.269 | |  | |  |  |  |  |  |  |  |  | 0.233 | |
| Well or fairly differentiated | 150 |  | 9 (60.0) |  | 126 (55.5) |  | 15 (41.7) |  |  | |  | | 19 |  | 9 (60.0) |  | 5 (33.3) |  | 5 (33.3) |  |  | |
| Poorly/undifferentiated/unknown | 128 |  | 6 (40.0) |  | 101 (44.5) |  | 21 (58.3) |  |  | |  | | 26 |  | 6 (40.0) |  | 10 (66.7) |  | 10 (66.7) |  |  | |
| *Tumor size (mm)* |  |  |  |  |  |  |  |  | 0.827 | |  | |  |  |  |  |  |  |  |  | 1.000* | |
| < 41 | 92 |  | 7 (46.7) |  | 73 (32.2) |  | 12 (33.3) |  |  | |  | | 20 |  | 7 (46.7) |  | 6 (40.0) |  | 7 (46.7) |  |  | |
| ≥ 41 | 99 |  | 5 (33.3) |  | 81 (35.6) |  | 13 (36.1) |  |  | |  | | 15 |  | 5 (33.3) |  | 5 (33.3) |  | 5 (33.3) |  |  | |
| Unknown | 87 |  | 3 (20.0) |  | 73(32.2) |  | 11 (30.6) |  |  | |  | | 10 |  | 3 (20.0) |  | 4 (26.7) |  | 3 (20.0) |  |  | |
| *Clinical stage (AJCC 2002)* |  |  |  |  |  |  |  |  | 0.481 | |  | |  |  |  |  |  |  |  |  | 0.122 | |
| Stage I-II | 126 |  | 8 (53.3) |  | 99 (43.6) |  | 19 (52.8) |  |  | |  | | 15 |  | 8 (53.3) |  | 3 (20.0) |  | 4 (26.7) |  |  | |
| Stage III | 152 |  | 7 (46.7) |  | 128 (56.4) |  | 17 (47.2) |  |  | |  | | 30 |  | 7 (46.7) |  | 12 (80.0) |  | 11 (73.3) |  |  | |
| *T stage* |  |  |  |  |  |  |  |  | 0.237* | |  | |  |  |  |  |  |  |  |  | 0.107* | |
| T_1-2_ | 83 |  | 6 (40.0) |  | 63 (27.8) |  | 14 (38.9) |  |  | |  | | 9 |  | 6 (40.0) |  | 2 (13.3) |  | 1 (6.7) |  |  | |
| T_3-4_ | 195 |  | 9 (60.0) |  | 164 (72.2) |  | 22 (61.1) |  |  | |  | | 36 |  | 9 (60.0) |  | 13 (86.7) |  | 14 (93.3) |  |  | |
| *N stage* |  |  |  |  |  |  |  |  | 0.083 | |  | |  |  |  |  |  |  |  |  | 0.537 | |
| Negative | 130 |  | 9 (60.0) |  | 99 (43.6) |  | 22 (61.1) |  |  | |  | | 22 |  | 9 (60.0) |  | 6 (40.0) |  | 7 (46.7) |  |  | |
| Positive | 148 |  | 6 (40.0) |  | 128 (56.4) |  | 14 (38.9) |  |  | |  | | 23 |  | 6 (40.0) |  | 9 (60.0) |  | 8 (53.3) |  |  | |

^‡^: 12 (80.0%) patients in the triple therapy group underwent at least partial esophagectomy, and one patient had excisional biopsy, but the surgical treatment of the other two patients was not detailed in the records; *: Fisher’s exact test.
