# Supplementary material for: Long-Term Survival Outcomes and Comparison of Different Treatment Modalities for Stage I-III Cervical Esophageal Carcinoma
Source: Front Med (Lausanne). 2021 Sep 22;8:714619. doi: 10.3389/fmed.2021.714619 (PMC8492900; doi:10.3389/fmed.2021.714619)
Supplement: Supplementary file 3 [file Table_3.docx]

Table S3. Univariate and multivariate analyses of overall survival (OS) for CEC patients who received different treatment modalities before and after propensity score matching (PSM) analysis.

|  | Overall survival (Before PSM) | | | | | | | | |  | Overall survival (After PSM) | | | | | | | | |
| --- | --- | --- | --- | --- | --- | --- | --- | --- | --- | --- | --- | --- | --- | --- | --- | --- | --- | --- | --- |
|  | Univariate | | | |  | Multivariate | | | |  | Univariate | | | |  | Multivariate | | | |
|  | ***P*** *value* | HR | *95% CI* | *95% CI* |  | ***P*** *value* | HR | *95% CI* | *95% CI* |  | ***P*** *value* | HR | *95% CI* | *95% CI* |  | ***P*** *value* | HR | *95% CI* | *95% CI* |
| *Factor* |  |  | *Lower* | *Upper* |  |  |  | *Lower* | *Upper* |  |  |  | *Lower* | *Upper* |  |  |  | *Lower* | *Upper* |
| *Age at diagnosis (years)* |  |  |  |  |  |  |  |  |  |  |  |  |  |  |  |  |  |  |  |
| < 65 | Reference |  |  |  |  | Reference |  |  |  |  | Reference |  |  |  |  | - |  |  |  |
| ≥ 65 | < 0.001 | 1.797 | 1.357 | 2.380 |  | < 0.001 | 1.727 | 1.296 | 2.301 |  | 0.273 | 1.479 | 0.735 | 2.976 |  |  |  |  |  |
| *Marital status* |  |  |  |  |  |  |  |  |  |  |  |  |  |  |  |  |  |  |  |
| Married | Reference |  |  |  |  | - |  |  |  |  | Reference |  |  |  |  | - |  |  |  |
| Unmarried and others | 0.131 | 1.242 | 0.937 | 1.646 |  |  |  |  |  |  | 0.387 | 1.332 | 0.696 | 2.546 |  |  |  |  |  |
| *Race* |  |  |  |  |  |  |  |  |  |  |  |  |  |  |  |  |  |  |  |
| White | Reference |  |  |  |  | - |  |  |  |  | Reference |  |  |  |  | - |  |  |  |
| Nonwhite | 0.142 | 1.265 | 0.924 | 1.733 |  |  |  |  |  |  | 0.962 | 0.981 | 0.447 | 2.151 |  |  |  |  |  |
| *Sex* |  |  |  |  |  |  |  |  |  |  |  |  |  |  |  |  |  |  |  |
| Female | Reference |  |  |  |  | Reference |  |  |  |  | Reference |  |  |  |  | - |  |  |  |
| Male | < 0.001 | 1.806 | 1.326 | 2.458 |  | < 0.001 | 1.776 | 1.298 | 2.431 |  | 0.607 | 1.241 | 0.545 | 2.829 |  |  |  |  |  |
| *Histology* |  |  |  |  |  |  |  |  |  |  |  |  |  |  |  |  |  |  |  |
| SCC | Reference |  |  |  |  | - |  |  |  |  | Reference |  |  |  |  | - |  |  |  |
| Non-SCC | 0.237 | 0.681 | 0.360 | 1.287 |  |  |  |  |  |  | 0.704 | 0.817 | 0.289 | 2.315 |  |  |  |  |  |
| *Differentiation* |  |  |  |  |  |  |  |  |  |  |  |  |  |  |  |  |  |  |  |
| Well or fairly differentiated | Reference |  |  |  |  | - |  |  |  |  | Reference |  |  |  |  | - |  |  |  |
| Poorly/undifferentiated/Unknown | 0.140 | 0.810 | 0.613 | 1.072 |  |  |  |  |  |  | 0.821 | 0.927 | 0.482 | 1.783 |  |  |  |  |  |
| *Tumor size (mm)* |  |  |  |  |  |  |  |  |  |  |  |  |  |  |  |  |  |  |  |
| < 41 | Reference |  |  |  |  | Reference |  |  |  |  | Reference |  |  |  |  | - |  |  |  |
| ≥ 41 | 0.017 | 1.515 | 1.077 | 2.129 |  | 0.062 | 1.388 | 0.983 | 1.959 |  | 0.801 | 1.097 | 0.532 | 2.264 |  |  |  |  |  |
| Unknown | 0.214 | 1.248 | 0.880 | 1.772 |  | 0.454 | 1.145 | 0.803 | 1.635 |  | 0.976 | 1.014 | 0.419 | 2.452 |  |  |  |  |  |
| *Clinical stage (AJCC 2002)* |  |  |  |  |  |  |  |  |  |  |  |  |  |  |  |  |  |  |  |
| Stage I-II | Reference |  |  |  |  | - |  |  |  |  | Reference |  |  |  |  | Reference |  |  |  |
| Stage III | 0.601 | 1.077 | 0.816 | 1.422 |  |  |  |  |  |  | 0.028 | 2.230 | 1.090 | 4.560 |  | 0.217 | 1.912 | 0.684 | 5.347 |
| *T stage* |  |  |  |  |  |  |  |  |  |  |  |  |  |  |  |  |  |  |  |
| T_1-2_ | Reference |  |  |  |  | - |  |  |  |  | Reference |  |  |  |  | Reference |  |  |  |
| T_3-4_ | 0.602 | 0.924 | 0.688 | 1.242 |  |  |  |  |  |  | 0.031 | 2.650 | 1.095 | 6.412 |  | 0.541 | 1.463 | 0.432 | 4.962 |
| *N stage* |  |  |  |  |  |  |  |  |  |  |  |  |  |  |  |  |  |  |  |
| Negative | Reference |  |  |  |  | - |  |  |  |  | Reference |  |  |  |  | - |  |  |  |
| Positive | 0.076 | 1.288 | 0.974 | 1.704 |  |  |  |  |  |  | 0.501 | 1.248 | 0.654 | 2.380 |  |  |  |  |  |
| *Treatment modality* |  |  |  |  |  |  |  |  |  |  |  |  |  |  |  |  |  |  |  |
| Single therapy | Reference |  |  |  |  | Reference |  |  |  |  | Reference |  |  |  |  | - |  |  |  |
| Double therapy | < 0.001 | 0.421 | 0.290 | 0.613 |  | < 0.001 | 0.471 | 0.321 | 0.689 |  | 0.281 | 0.647 | 0.294 | 1.428 |  | 0.128 | 0.516 | 0.220 | 1.209 |
| Triple therapy | 0.002 | 0.350 | 0.181 | 0.679 |  | 0.008 | 0.399 | 0.202 | 0.789 |  | 0.028 | 0.407 | 0.182 | 0.909 |  | 0.038 | 0.410 | 0.177 | 0.951 |

HR, hazard ratio; CI, confidence interval.
