# Supplementary figures and images for: Long-Term Survival Outcomes and Comparison of Different Treatment Modalities for Stage I-III Cervical Esophageal Carcinoma
Source: Front Med (Lausanne). 2021 Sep 22;8:714619. doi: 10.3389/fmed.2021.714619 (PMC8492900; doi:10.3389/fmed.2021.714619)

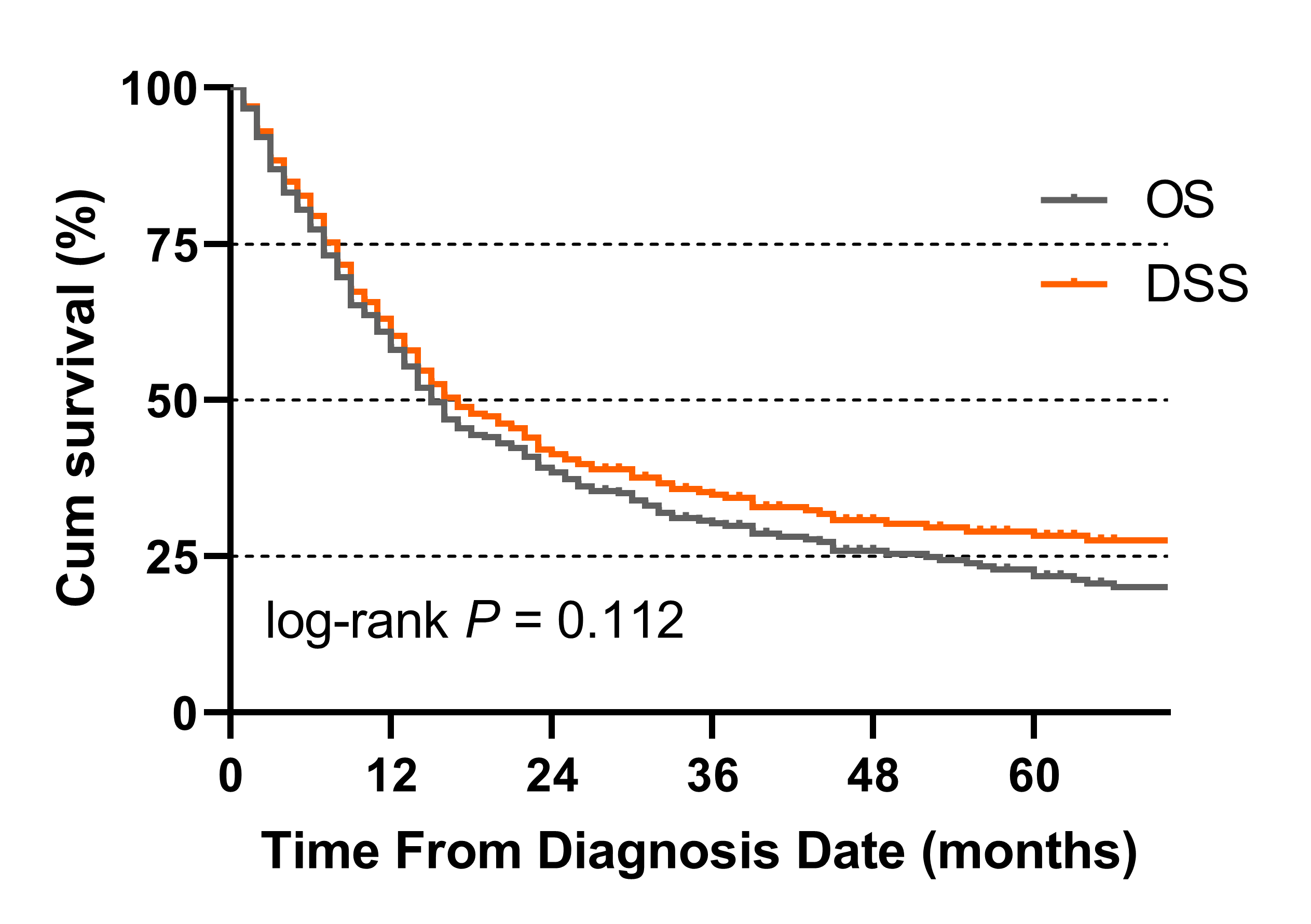

Supplement: Supplementary file 4 [file Image_1.TIF]
